# Contrast-enhanced cardiac MRI is superior to non-contrast mapping to predict left ventricular remodeling at 6 months after acute myocardial infarction

**DOI:** 10.1007/s00330-023-10100-9

**Published:** 2023-09-04

**Authors:** Hang Chen, Jennifer Erley, Kai Muellerleile, Dennis Saering, Charlotte Jahnke, Ersin Cavus, Jan N. Schneider, Stefan Blankenberg, Gunnar K. Lund, Gerhard Adam, Enver Tahir, Martin Sinn

**Affiliations:** 1 Department of Diagnostic and Interventional Radiology and Nuclear Medicine, University Hospital Hamburg Eppendorf, Martinistr. 52, 20246 Hamburg, Germany; 2 Department of General and Interventional Cardiology, University Heart Center, Hamburg, Germany; 3 German Center for Cardiovascular Research (DZHK), Partner Site Hamburg/Kiel/Lübeck, Hamburg, Germany; 4 Information Technology and Image Processing, University of Applied Sciences, Wedel, Germany

**Keywords:** Multiparametric magnetic resonance imaging, Myocardial infarction, Extracellular matrix, Ventricular remodeling

## Abstract

**Objectives:**

Parametric mapping constitutes a novel cardiac magnetic resonance (CMR) technique enabling quantitative assessment of pathologic alterations of left ventricular (LV) myocardium. This study aimed to investigate the clinical utility of mapping techniques with and without contrast agent compared to standard CMR to predict adverse LV remodeling following acute myocardial infarction (AMI).

**Materials and methods:**

A post hoc analysis was performed on sixty-four consecutively enrolled patients (57 ± 12 years, 54 men) with first-time reperfused AMI. Baseline CMR was obtained at 8 ± 5 days post-AMI, and follow-up CMR at 6 ± 1.4 months. T1/T2 mapping, T2-weighted, and late gadolinium enhancement (LGE) acquisitions were performed at baseline and cine imaging was used to determine adverse LV remodeling, defined as end-diastolic volume increase by 20% at 6 months.

**Results:**

A total of 11 (17%) patients developed adverse LV remodeling. At baseline, patients with LV remodeling showed larger edema (30 ± 11 vs. 22 ± 10%LV; *p* < 0.05), infarct size (24 ± 11 vs. 14 ± 8%LV; *p* < 0.001), extracellular volume (ECV_infarct_; 63 ± 12 vs. 47 ± 11%; *p* < 0.001), and native T2_infarct_ (95 ± 16 vs. 78 ± 17 ms; *p* < 0.01). ECV_infarct_ and infarct size by LGE were the best predictors of LV remodeling with areas under the curve (AUCs) of 0.843 and 0.789, respectively (all *p* < 0.01). Native T1_infarct_ had the lowest AUC of 0.549 (*p* = 0.668) and was inferior to edema size by T2-weighted imaging (AUC = 0.720; *p* < 0.05) and native T2_infarct_ (AUC = 0.766; *p* < 0.01).

**Conclusion:**

In this study, ECV_infarct_ and infarct size by LGE were the best predictors for the development of LV remodeling within 6 months after AMI, with a better discriminative performance than non-contrast mapping CMR.

**Clinical relevance statement:**

This study demonstrates the predictive value of contrast-enhanced and non-contrast as well as conventional and novel CMR techniques for the development of LV remodeling following AMI, which might help define precise CMR endpoints in experimental and clinical myocardial infarction trials.

**Key Points:**

*• Multiparametric CMR provides insights into left ventricular remodeling at 6 months following an acute myocardial infarction.*

*• Extracellular volume fraction and infarct size are the best predictors for adverse left ventricular remodeling.*

*• Contrast-enhanced T1 mapping has a better predictive performance than non-contrast standard CMR and T1/T2 mapping.*

**Supplementary Information:**

The online version contains supplementary material available at 10.1007/s00330-023-10100-9.

## Introduction

Left ventricular (LV) remodeling is a possible complication after an acute myocardial infarction (AMI). Adverse LV remodeling after AMI is defined as a progressive increase of LV end-diastolic volume ≥ 20% at 6 months following AMI [1]. LV enlargement and architectural alterations develop in response to a loss of contractile myocardium and increased wall stress [2, 3]. However, the pathophysiology of LV remodeling following AMI is not entirely understood. LV remodeling is a known and significant risk factor for adverse prognosis [4, 5].

Cardiac magnetic resonance (CMR) imaging has greatly improved the understanding of the cardiac function and tissue characteristics after AMI, providing incremental information for predicting LV remodeling and adverse cardiovascular events [6]. Late gadolinium enhancement (LGE) imaging by CMR is a well-validated approach for quantifying myocardial infarction [7], yet possibly overestimates myocardial infarct size in the acute phase compared with histopathology due to severe edema as validated in animal models [8, 9]. Recently, novel parametric CMR mapping techniques have evolved and enable quantitative tissue characterization in the infarcted and remote myocardium [10]. T2 relaxation time allows a reliable estimation of myocardial edema and is a robust alternative to conventional T2-weighted (T2w) edema imaging [11]. This technique has already been used to determine the area of myocardial injury [12] and to assess infarction chronicity [13]. Furthermore, T1 mapping facilitates the estimation of extracellular volume (ECV) thus allowing the differentiation of reversible from irreversible myocardial injury after reperfusion [14].

Although multiparametric CMR characterization of damaged myocardium following AMI has been done, there are limited data on the utility of CMR parameters for predicting adverse LV remodeling in the chronic phase of myocardial infarction. Therefore, this single-center study aimed to investigate the clinical utility of mapping techniques with or without contrast agent compared to standard CMR to predict adverse LV remodeling following AMI.

## Materials and methods

### Study population

This ambispective single-center study was approved by the local ethics committee and complied with the Declaration of Helsinki; all subjects gave their written informed consent. Consecutive patients with a first reperfused AMI through primary percutaneous coronary intervention were enrolled from April 2011 to August 2016. Patients underwent baseline (8 ± 5 days) and follow-up (6 ± 1.4 months) CMR after AMI, including both ST-segment elevation myocardial infarct (STEMI) and non-ST-segment elevation myocardial infarct (NSTEMI). AMI was defined according to established criteria [15]. Exclusion criteria were previous AMI or coronary artery bypass grafting, severe renal failure (estimated glomerular filtration rate [eGFR] < 30 mL/min/1.73 m^2^), persisting atrial fibrillation or CMR contraindications. This post hoc analysis was conducted in 64 consecutively recruited patients. Furthermore, the study population partly consisted of individuals, who had been evaluated in two previously published studies, one focused on the diagnostic accuracy of quantitative native T1 and T2 mapping compared to the assessment of edema on standard T2w imaging to differentiate acute from chronic MI [13], the other demonstrated the development of global and segmental myocardial strain in acute and chronic STEMI [16].

### Image acquisition

CMR acquisitions were performed on a 1.5-Tesla MR scanner (Achieva, Philips Medical Systems), equipped with a 5-channel cardiac coil, and all sequences were electrocardiographically triggered. The imaging protocol included standard balanced steady-state free-precession (SSFP) cine imaging in short-axis, 2-, 3-, and 4-chamber views and edema-sensitive black T2w imaging by a fat-suppressed triple inversion-recovery sequence with the following parameters: voxel size 1.36 × 1.36 × 6 mm^3^, echo time (TE) = 1.67 ms, time to repetition (TR) = 3.34 ms, flip angle (FA) = 60°. T1 mapping was performed using a modified look-locker inversion recovery (MOLLI) sequence with the following parameters: voxel size 1.72 × 1.72 × 10 mm^3^, TE = 1.05 ms, TR = 2.58 ms, FA = 35°, linear phase encoding, 8 single-shot balanced SSFP readouts, typical effective inversion times between 150 and 3871 ms [17]. T2 mapping was performed with a free-breathing navigator-gated black-blood prepared gradient and spin-echo hybrid sequence in three LV end-diastolic short-axis (basal, mid, and apical) slices, corresponding to the MOLLI sequence, with the following parameters: voxel size 1.05 × 1.05 × 10 mm^3^, TE = 12.5–62.4 ms, TR = 1600 ms, FA = 90°, 9 readouts [18]. After administration of 0.075 mmol/kg gadobenate dimeglumine (MultiHance, Bracco), end-diastolic LGE imaging was acquired using an end-diastolic phase-sensitive inversion recovery sequence [13].

### CMR data analysis

**Fig. 1:**
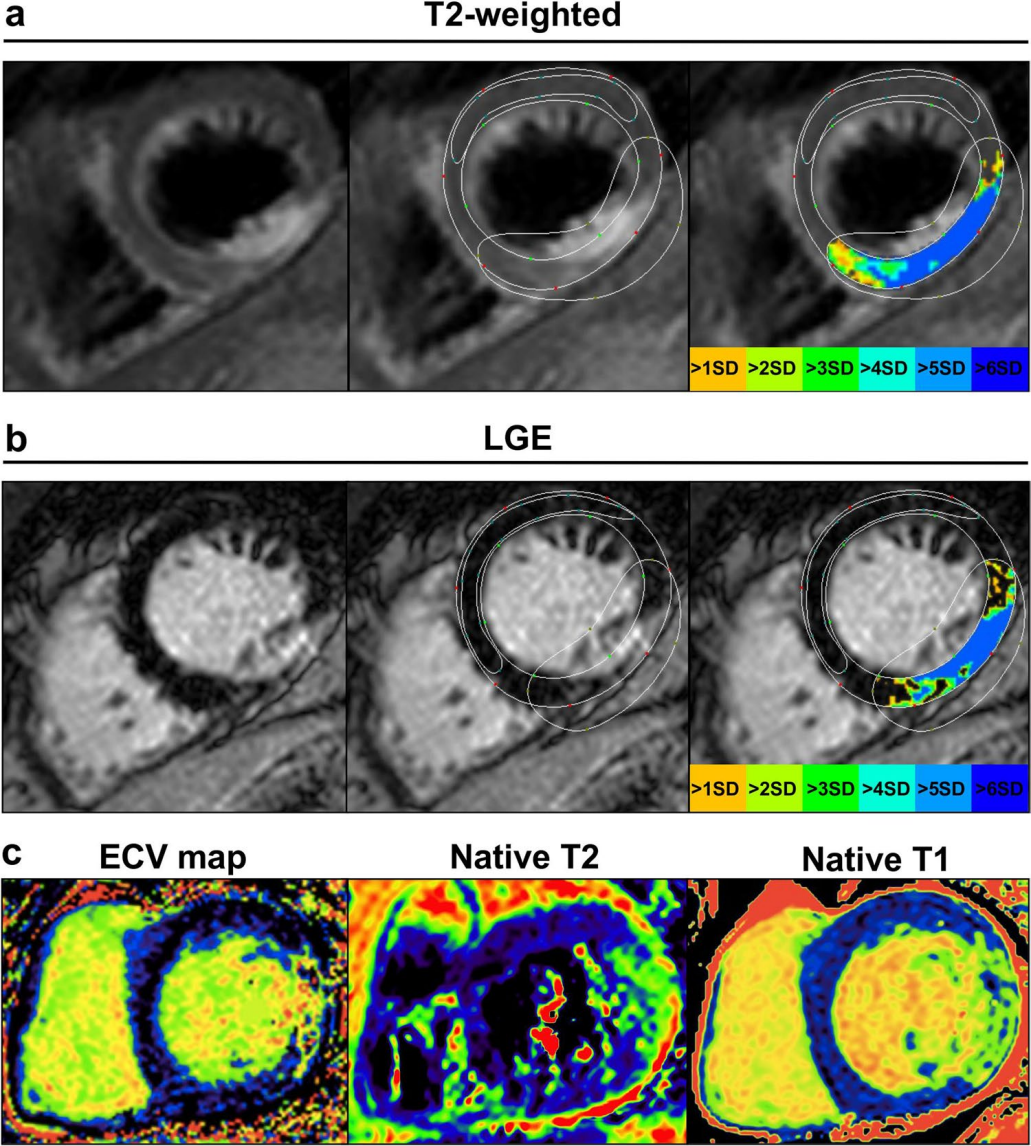
Cardiac T2-weighted CMR image (**a**), LGE image (**b**), ECV, native T2 and T1 maps (**c**). For quantitative analysis of lesion size, a large region of interest (ROI) was placed in remote myocardium to quantify mean signal intensity or mean relaxation times, including standard deviations as shown in **a** and **b**. The threshold was set at >2 SDs to define lesion size in the infarct area. Care was taken not to include the blood volume or the epicardial fat in the measurements by precise placement of the endo- and epicardial myocardial borders, as shown in the colored T2-weighted and LGE images. ECV, extracellular volume; LGE, late gadolinium enhancement; SD, standard deviation

Two experienced radiologists (E.T. and M.S. with 9 and 6 years of CMR experience) independently and blindly analyzed each CMR in random order using a commercially available software (CVi42, Circle Cardiovascular Imaging Inc.) and an in-house developed dedicated software [19]. Parameters were indexed to the body surface area. Evaluation of LV indexed end-diastolic volume (EDVi), end-systolic volume (ESVi), and myocardial mass was performed in standard fashion on short-axis cine images [20]. ECV and native T2 and T1 maps were generated using a plug-in for OsiriX software (Pixmeo). ECV was calculated using native and post-contrast T1 maps [21]. Meticulous care was taken to delineate endo- and epicardial contours with 10% endo- and epicardial offsets to avoid contamination [13]. LGE areas were visually identified and the signal intensity of edematous or infarcted myocardium was set as >2 standard deviations (SDs) of remote myocardium [13, 22]. Regions of interest (ROIs) were placed in the infarcted area using LGE as a reference. Infarcted and edematous area refers to the percentage of the enhanced myocardium above the threshold against the total area (%LV), which was calculated by dividing this area by the total amount of the LV area on the three short-axis sections [13, 23]. ECV and native T1 and T2 were measured in the infarcted and remote areas respectively. Figure 1 displays an example of ROI delineation. In addition, the infarcted and edematous area, native T2 and T1 relaxation times, and ECV of the involved segments were analyzed according to the American Heart Association (AHA) 16-segment model.

### Definition of adverse LV remodeling after AMI

The presence of adverse LV remodeling was defined as a progressive increase of indexed LVEDV ≥ 20% at 6 months following AMI compared to baseline according to previous literature [1].

### Statistical analysis

Calculations and graphics were performed with SPSS (version 28.0, IBM SPSS Statistics) and GraphPad Prism (version 9.2.0, GraphPad Software, LLC.). Continuous variables are expressed as mean ± SD or median (interquartile range). Comparisons for continuous data used the independent samples *t*-test or Mann-Whitney *U* test as appropriate. Categorical data are presented as absolute numbers (percentage) and were compared using *χ*^2^ test or Fischer’s exact test as appropriate. A receiver-operating characteristic (ROC) curve analysis was performed to assess the predictive performance by calculating the area under the curve (AUC) and optimal cutoff values from the ROC curves using the Youden’s index. Quantitative measurements were correlated with the Pearson coefficient. Statistical significance was defined as two-tailed *p* < 0.05.

## Results

### Patient characteristics

A total of 64 patients (57 ± 12 years, range 32–79 years, 84% men), who suffered AMI (STEMI: *n* = 34; NSTEMI: *n* = 30), were included in the final study cohort and underwent both baseline and 6-month follow-up CMR, of whom 11 (17%) developed adverse LV remodeling. Table 1 gives anthropometrics and clinical characteristics of AMI patients. There were no significant differences in anthropometrics, cardiovascular risk factors, and cardiac medications (*p *> 0.05 for all comparisons) between patients with and without adverse LV remodeling. Patients with subsequent adverse LV remodeling had higher peak creatine kinase (CK) with 2613 U/L (Q1–Q3: 1247–4711 U/L) than patients without remodeling with 788 U/L (Q1–Q3: 229–1742 U/L; *p* < 0.01). Right coronary artery–related infarct was more frequent in patients without remodeling (87% vs. 36%; *p* < 0.01), whereas the incidence of infarct in left anterior descending artery (*p* = 0.331) and circumflex artery (*p* = 0.445) was similar (Table 1).

**Table 1:** Anthropometrics and clinical characteristics in patients following an acute myocardial infarction with and without development of adverse LV remodeling at 6-month follow-up

	All patients (*n* = 64)	No LV remodeling (*n* = 53)	LV remodeling (*n* = 11)	*p-*value
Anthropometrics				
Age, years	57 ± 12	57 ± 12	57 ± 10	0.963
Male sex, *n* (%)	54 (84)	44 (83)	10 (91)	0.512
BSA, m^2^	2.04 ± 0.20	2.05 ± 0.21	1.97 ± 0.15	0.199
Risk factors				
Smoking, *n* (%)	41(64)	33 (62)	8 (73)	0.732
Diabetes, *n* (%)	8 (12.5)	6 (11)	2 (18)	0.617
Hypertension, *n* (%)	38 (59)	29 (55)	9 (82)	0.178
Hyperlipidemia, *n* (%)	22 (34)	19 (36)	3 (27)	0.735
Family history, *n* (%)	21 (33)	17 (32)	4 (36)	0.999
AMI type				
STEMI, *n* (%)	34 (53)	26 (49)	8 (73)	0.152
NSTEMI, *n* (%)	30 (47)	27 (51)	3 (27)	-
Cardiac enzymes				
Troponin T, pg/mL	1643 (517–4285)	1450 (442–3619)	3179 (1395–6210)	0.121
Peak CK, U/L	881 (257–2319)	788 (229–1742)	2613 (1247–4711)	** < 0.01**
Peak CK-MB, U/L	102 (39–274)	93 (38–268)	209 (79–413)	0.225
Infarct-related artery				
LAD, *n* (%)	27 (42)	24 (45)	3 (27)	0.331
CFX, *n* (%)	16 (25)	12 (23)	4 (36)	0.445
RCA, *n* (%)	50 (78)	46 (87)	4 (36)	** < 0.01**
Cardiac medications				
ACEI or ARB, *n* (%)	16 (25)	12 (23)	4 (36)	0.445
Beta-blocker, *n* (%)	17 (27)	13 (25)	4 (36)	0.463
Diuretics, *n* (%)	9 (14)	8 (15)	1 (9)	0.999
Statins, *n* (%)	10 (16)	8 (15)	2 (18)	0.999
Aspirin/Clopidogrel, *n* (%)	15 (23)	12 (23)	3 (27)	0.710
Calcium antagonists, *n* (%)	6 (9)	4 (8)	2 (18)	0.271

Variables are presented as mean ± SD or median (interquartile range) for continuous data and n (%) for categorical data

Values in bold denote significant differences between groups

*Abbreviations*: *ACEI* angiotensin-converting enzyme inhibitor, *AMI* acute myocardial infarct, *ARB* angiotensin receptor blocker, *BSA* body surface area, *CFX* circumflex artery, *CK* creatine kinase, *CK-MB* creatine kinase myocardial band, *LAD* left anterior descending artery, *NSTEMI* non-ST-segment elevation myocardial infarct, *RCA* right coronary artery, *STEMI* ST-segment elevation myocardial infarct

### CMR findings in patients with and without LV remodeling at baseline

**Fig. 2:**
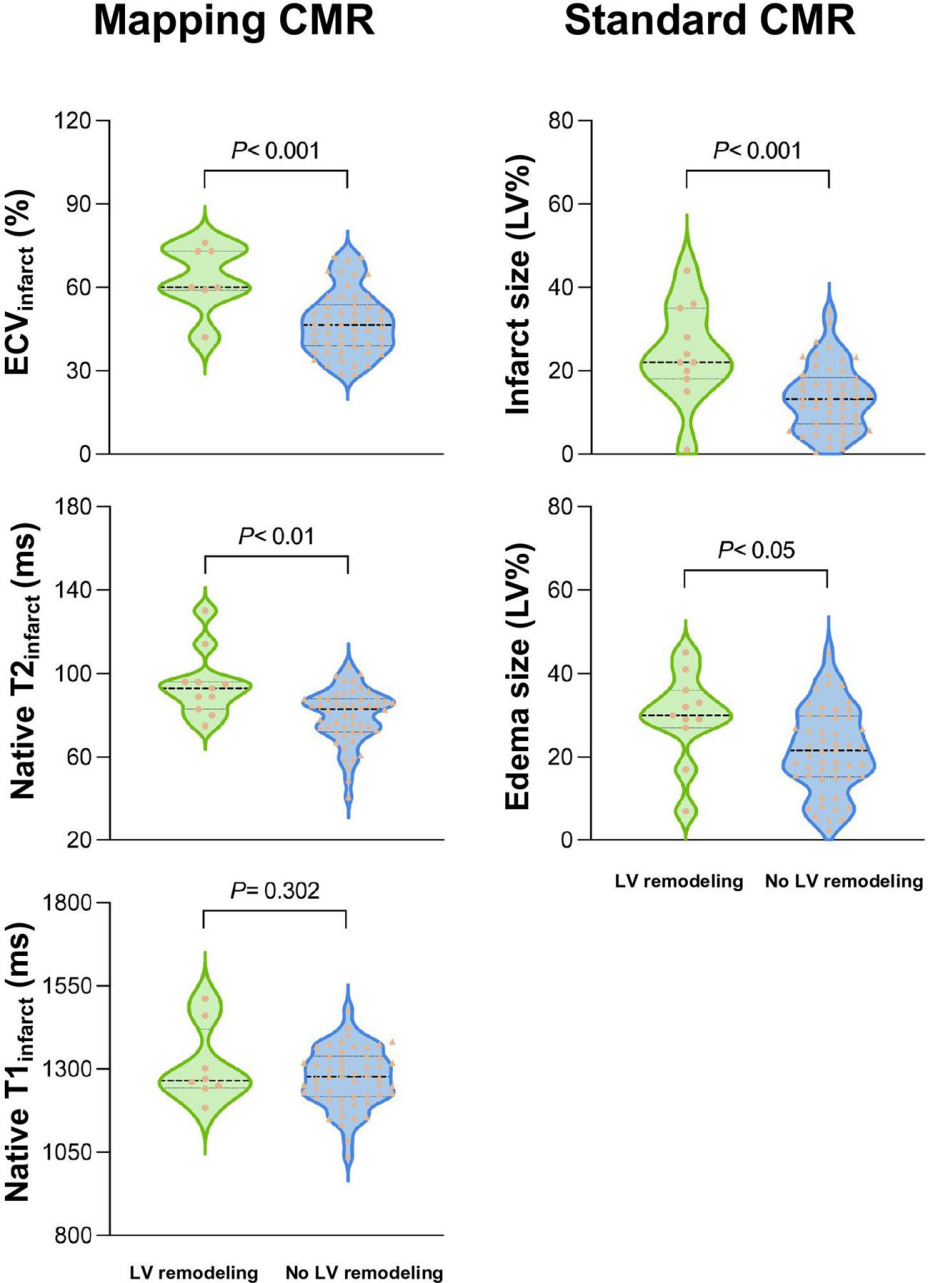
Comparisons of mapping and standard CMR parameters at baseline between patients with and without adverse LV remodeling. Mapping CMR at baseline (left column) shows patients who developed adverse LV remodeling at 6 months had significantly higher ECV_infarct_ and native T2_infarct_ but similar native T1_infarct_. Standard CMR at baseline (right column) shows patients who developed adverse LV remodeling had larger edema size and infarct size than those without remodeling. ECV, extracellular volume; LV, left ventricular

At baseline, edema size (30 ± 1%LV vs. 22 ± 10%LV; *p* < 0.05) and infarct size (24 ± 11%LV vs. 14 ± 8 %LV; *p* < 0.001) were larger in AMI patients, who developed adverse LV remodeling (Table 2; Fig. 2). LV functional and morphological parameters were similar in the two groups. At the acute stage, patients with adverse LV remodeling showed higher ECV_infarct_ (63 ± 12% vs. 47 ± 11%; *p* < 0.001) and native T2_infarct_ (95 ± 16 ms vs. 78 ± 17 ms; *p* < 0.01), but there was no difference in native T1_infarct_ (*p* = 0.302) (Fig. 2). ECV_remote_, native T1_remote_, and native T2_remote_ did not differ between patients with and without LV remodeling (*p *> 0.05 for all comparisons; Table 2). Figures S1–5 represent the involved segments with the respective infarcted and edematous area, native T2 and T1 relaxation times, and ECV in patients with and without adverse LV remodeling according to the AHA 16-segment model.

**Table 2:** Baseline and follow-up CMR parameters in patients following an acute myocardial infarction with and without development of adverse LV remodeling at 6-month follow-up

CMR parameters	All patients (*n* = 64)	No LV remodeling (*n* = 53)	LV remodeling (*n* = 11)	*p-*value
Baseline				
Standard CMR				
- Edema size, %LV	23 ± 11	22 ± 10	30 ± 11	** < 0.05**
- Infarct size, %LV	16 ± 9	14 ± 8	24 ± 11	** < 0.001**
- LVEF, %	56 ± 10	57 ± 9	53 ± 13	0.310
- LV mass index, g/m^2^	70 ± 15	69 ± 14	73 ± 19	0.525
- LVEDV, mL/m^2^	77 ± 12	77 ± 11	75 ± 14	0.608
- LVESV, mL/m^2^	34 ± 10	33 ± 8	36 ± 14	0.336
Mapping CMR				
- ECV_infarct_, %	49 ± 12	47 ± 11	63 ± 12	** < 0.001**
- ECV_remote_, %	27 ± 4	26 ± 4	28 ± 2	0.212
- Native T2_infarct_, ms	82 ± 15	80 ± 13	95 ± 16	** < 0.01**
- Native T2_remote_, ms	54 ± 4	53 ± 4	56 ± 3	0.170
- Native T1_infarct_, ms	1279 ± 92	1274 ± 88	1310 ± 114	0.302
- Native T1_remote_, ms	1040 ± 50	1039 ± 53	1050 ± 27	0.542
6-month follow-up				
Edema size, %LV	3 ± 2	3 ± 3	3 ± 2	0.961
Infarct size, %LV	11 ± 8	10 ± 7	16 ± 10	** < 0.05**
LVEF, %	57 ± 9	59 ± 8	50 ± 12	** < 0.01**
LV mass index, g/m^2^	63 ± 13	62 ± 12	68 ± 17	0.199
LVEDVi, mL/m^2^	80 ± 14	77 ± 11	95 ± 19	** < 0.001**
LVESVi, mL/m^2^	35 ± 13	31 ± 9	49 ± 20	** < 0.05**

Variables are presented as mean ± SD or median (interquartile range) for continuous data and *n* (%) for categorical data

Values in **bold** denote significant differences between groups

*Abbreviations*: *CMR* cardiovascular magnetic resonance, *ECV* extracellular volume, *LV* left ventricle, *LVEF* left ventricular ejection fraction, *LVEDVi* left ventricular end-diastolic volume index, *LVESVi* left ventricular end-systolic volume index

### CMR findings in patients with and without LV remodeling at 6-month follow-up

AMI patients with subsequent adverse LV remodeling had a larger infarct size (16 ± 10%LV vs. 10 ± 7%LV, *p* < 0.05) and reduced LVEF (50 ± 12% vs. 59 ± 8%; *p* < 0.01; Table 2). However, edema size was similar in both groups at 6-month follow-up. LVEDVi (95 ± 19 mL/m^2^ vs. 77 ± 11 mL/m^2^; *p* < 0.001) and LVESVi (49 ± 20 mL/m^2^ vs. 31 ± 9 mL/m^2^; *p* < 0.05) were higher in patients with LV remodeling, but LV mass index was similar (Table 2).

**Fig. 3:**
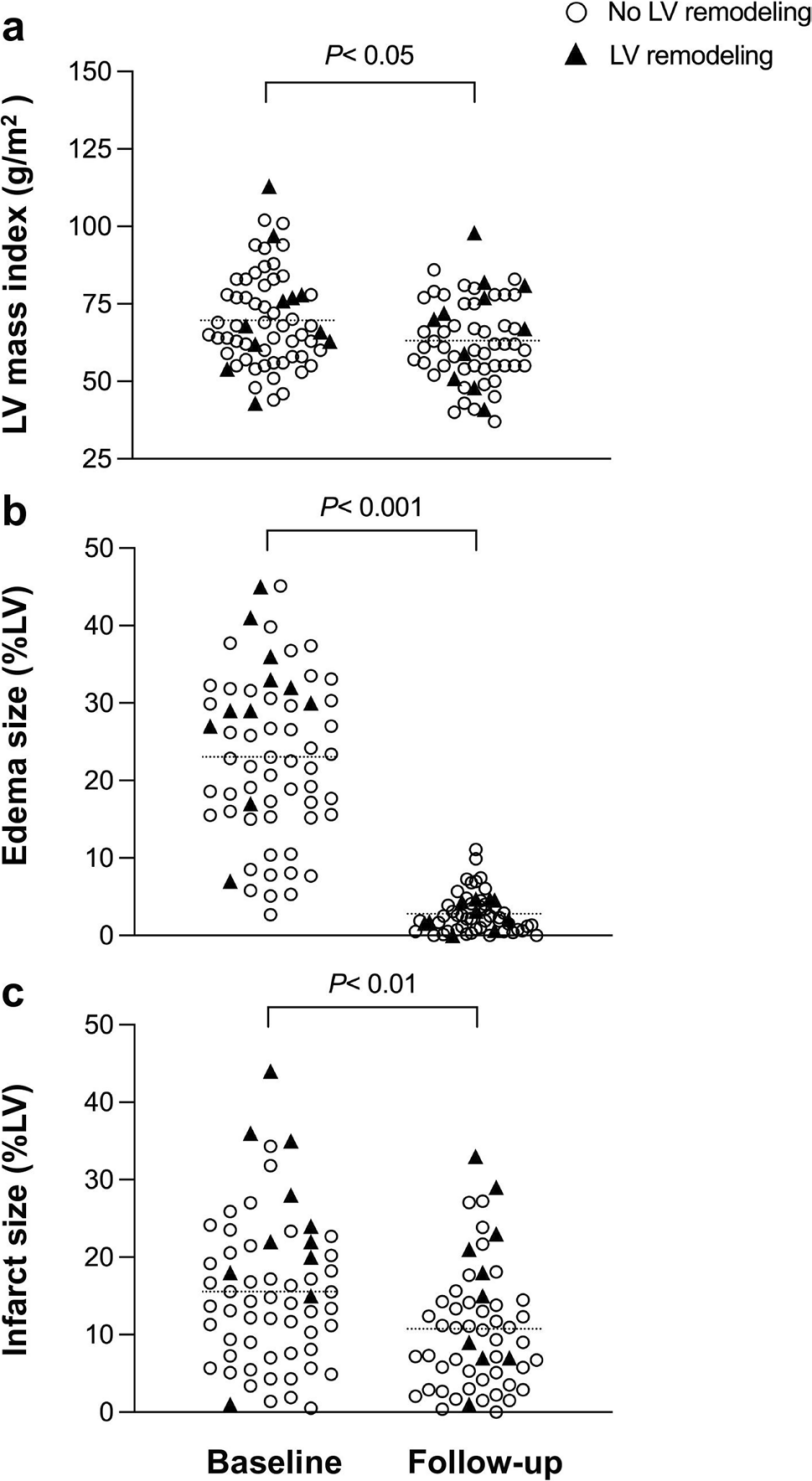
Changes of LV mass index (**a**), edema size (**b**), and infarct size (**c**) in patients with and without adverse LV remodeling at 6 months following AMI. AMI, acute myocardial infarct; LV, left ventricular

A longitudinal comparison of AMI patients showed that indexed LV mass decreased from 70 ± 15 g/m^2^ at baseline to 63 ± 13 g/m^2^ at 6-month follow-up (*p* < 0.05). Edema size (23 ± 11%LV vs. 3 ± 2%LV; *p* < 0.001) and infarct size (16 ± 9%LV vs. 11 ± 8%LV; *p* < 0.01) had markedly declined at 6 months (Fig. 3). AMI patients without LV remodeling showed that edema size (*p* < 0.001), infarct size (*p* < 0.01), and LV mass index (*p* < 0.01) significantly decreased at 6 months following AMI. No statistical differences were found in LVEF (*p* = 0.177), LVEDVi (*p* = 0.858), and LVESVi (*p* = 0.305). AMI patients with adverse remodeling showed decreased edema size (*p* < 0.001) and increased LVEDVi (*p* < 0.05) at follow-up compared to baseline, but infarct size (*p* = 0.124), LVEF (*p* = 0.511), LV mass index (*p* = 0.551), and LVESVi (*p* = 0.108) did not change.

### Predictive performance of multiparametric CMR and cardiac biomarkers

**Fig. 4:**
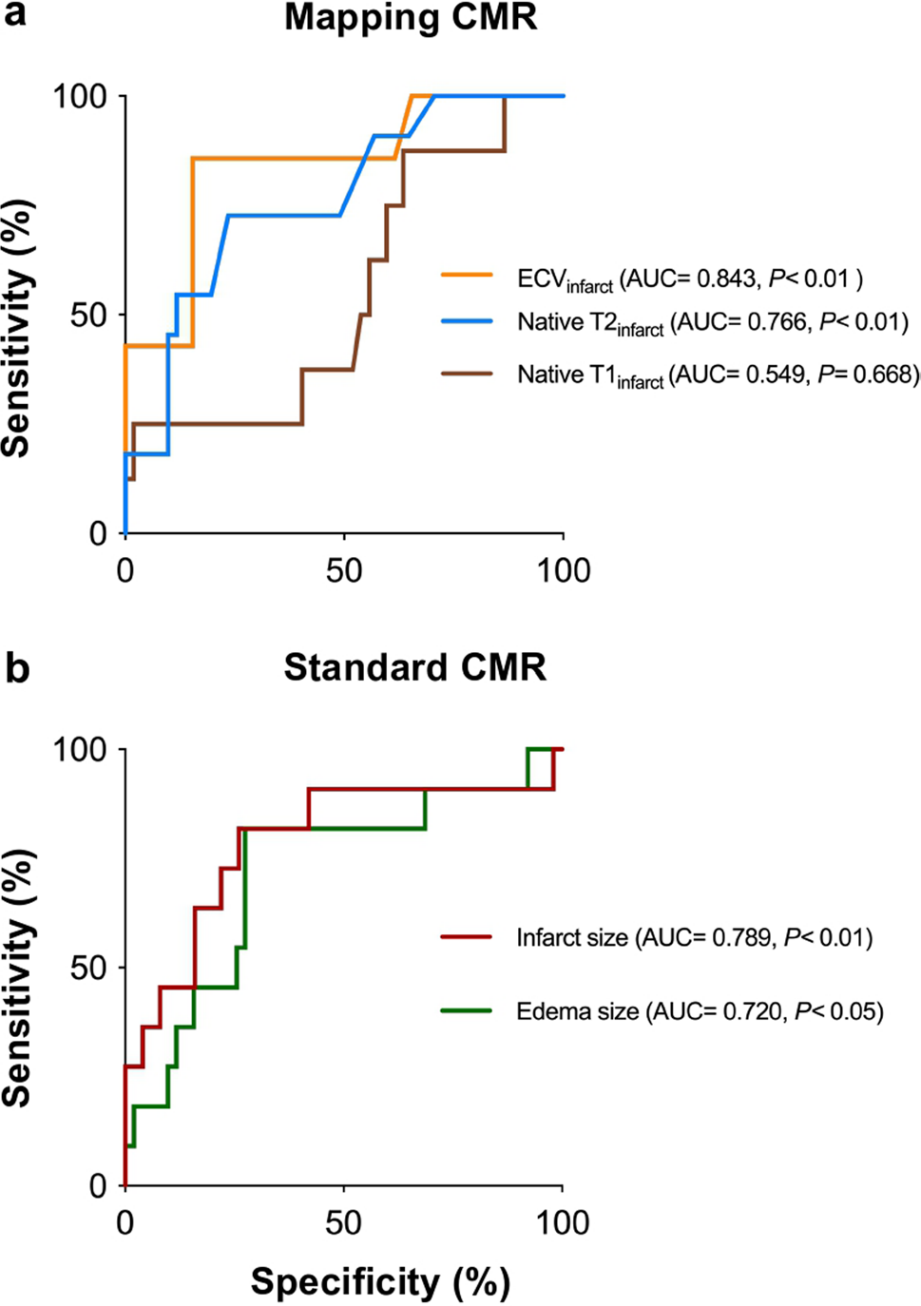
Receiver-operating characteristic curve analysis for the prediction of adverse LV remodeling at 6 months following AMI with mapping (**a**) and standard CMR (**b**). ECV_infarct_ and infarct size by LGE were the best predictors of adverse LV remodeling with AUCs of 0.843 (*p < *0.01) and 0.789 (*p < *0.01), respectively. Native T1_infarct_ had the lowest AUC of 0.549 (*p* = 0.668) and was inferior to edema size by T2-weighted (AUC = 0.720; *p* < 0.05) and native T2_infarct_ (AUC = 0.766; *p* < 0.01) in predicting adverse LV remodeling. AMI, acute myocardial infarct; AUC, area under the curve; ECV, extracellular volume; LV, left ventricular

ROC curve analysis for the prediction of adverse LV remodeling after AMI at 6-month follow-up revealed that ECV_infarct_ and infarct size by LGE were the best predictors with AUCs of 0.843 and 0.789 (*p* < 0.01 for both), respectively (Table 3 and Fig. 4). ECV_infarct_ resulted in good sensitivity (0.86; 95% CI: 0.47–0.99) and specificity (0.85; 95% CI: 0.72–0.92). Infarct size by LGE also had a good sensitivity of 0.82 (95% CI: 0.51–0.96) and specificity of 0.74 (95% CI: 0.60–0.84). Native T1_infacrt_ had the lowest AUC of 0.549 (*p* = 0.668) and was inferior to edema size by T2w (AUC = 0.720; *p* < 0.05) and native T2_infarct_ (AUC = 0.766; *p* < 0.01) in predicting adverse LV remodeling. Additionally, the predictive performance of CMR parameters in the involved segments based on the AHA bull’s eye model was also assessed by ROC curve analysis. The findings resulted in a cutoff value of 34% for ECV (AUC = 0.653; *p* < 0.01) and 66 ms for native T2 relaxation times (AUC = 0.686; *p* < 0.01) in the involved segments for predicting adverse LV remodeling at 6-month follow-up (Table S1). The AUCs of cardiac biomarkers were 0.762 (*p* < 0.01) for peak CK, and 0.624 (*p* = 0.269) for peak CK-MB (Table 3).

**Table 3:** Receiver-operating characteristic curve analysis of CMR parameters and cardiac biomarkers at baseline to predict adverse LV remodeling at 6-month follow-up

								Total	Sensitivity	Specificity	Accuracy	PPV	NPV
Parameters	AUC (95% CI)	*p*-value*	Cutoff	TP	FN	FP	TN	(*n* = 64)	(%)	(%)	(%)	(%)	(%)
ECV_infarct_	0.843 (0.725–0.925)	< 0.01	> 57%	6	1	8	44	59	86 (47–99)	85 (72–92)	85 (73–92)	43 (21–67)	98 (87–100)
Infarct size	0.789 (0.666–0.883)	< 0.01	> 17%	9	2	13	37	61	82 (51–96)	74 (60–84)	75 (63–85)	41 (23–61)	95 (82–99)
Native T2_infarct_	0.766 (0.641–0.864)	< 0.01	> 88 ms	8	3	12	39	62	73 (43–91)	76 (63–86)	76 (64–85)	40 (22–61)	93 (80–98)
Edema size	0.720 (0.592–0.827)	< 0.05	> 27%	9	2	14	37	62	82 (51–96)	73 (59–83)	74 (62–84)	39 (22–59)	95 (82–99)
Native T1_infarct_	0.549 (0.415–0.678)	0.668	> 1236 ms	7	1	33	19	60	88 (47–100)	37 (24–51)	43 (32–56)	18 (8–32)	95 (75–100)
Peak CK	0.762 (0.639–0.859)	< 0.01	> 1109 U/L	9	2	18	35	64	82 (48–98)	66 (52–79)	69 (57–79)	33 (19–52)	95 (81–99)
Peak CK–MB	0.624 (0.482–0.752)	0.269	> 168 U/L	7	3	15	29	54	70 (35–93)	66 (50–80)	67 (53–78)	32 (16–53)	91 (75–98)

^*^For statistical significance of AUCs

*Abbreviations*: *AUC* area under the curve, *CI* confidence interval, *CK* creatine kinase, *CK-MB* creatine kinase myocardial band, *ECV* extracellular volume, *FN* false negative, *FP* false positive, *NPV* negative predictive value, *PPV* positive predictive value, *TN* true negative, *TP* true positive

### Correlations of mapping and standard CMR with the change in LVEDVi

**Fig. 5:**
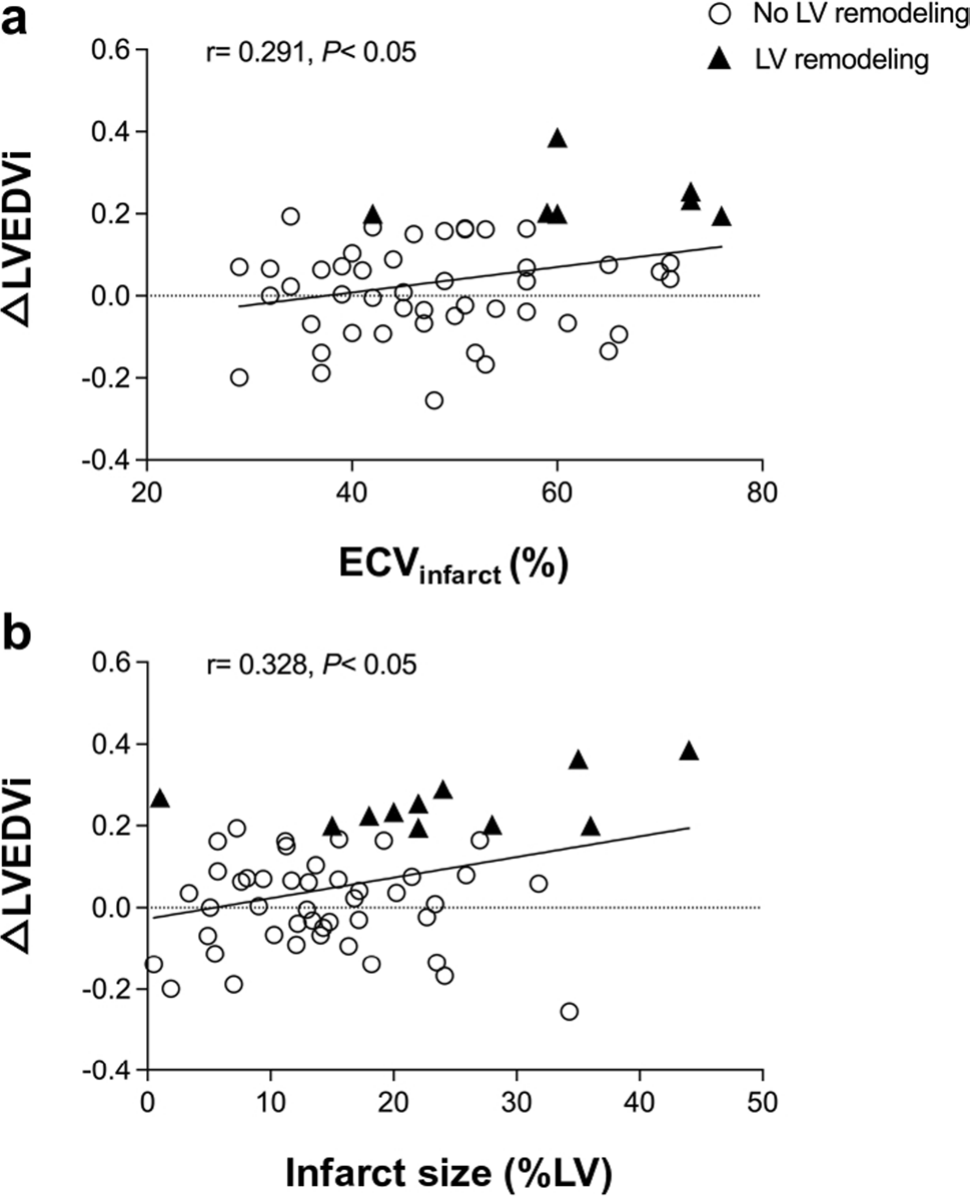
Correlation of ECV_infarct_ and infarct size at baseline with change in LVEDVi at 6 months following AMI. △LVEDVi is calculated by dividing the change in LVEDVi by initial LVEDVi. A positive correlation was found between ECV_infarct_ (**a**) and infarct size (**b**) at baseline and △LVEDVi. Variables above the horizontal dashed line indicate the increase in LVEDVi; conversely, below the horizontal dashed line indicates the decrease in LVEDVi at 6 months after AMI. Triangles represent these patients developed adverse LV remodeling, who had an obvious tendency in higher ECV_infarct_ and larger infarct size as well as significant increase in LVEDVi. AMI, acute myocardial infarct; ECV, extracellular volume; LVEDVi, left ventricular end-diastolic volume index

ECV_infarct_ (*r* = 0.291; *p* < 0.05) and infarct size assessed by LGE at baseline (*r* = 0.328; *p* < 0.05) had a positive association with the change in LVEDVi at 6 months after AMI. As shown in Fig. 5, patients with development of adverse LV remodeling had an obvious tendency to higher ECV_infarct_ and larger infarct size, and a significant increase in LVEDVi. The change in LVEDVi also correlated with native T2_infarct_ (*r* = 0.333; *p* < 0.05) and edema size (*r* = 0.283; *p* < 0.05) at baseline, but not with native T1_infarct_ (*p* = 0.103).

## Discussion

In this study, we analyzed the predictive performance of multiparametric mapping techniques compared to standard CMR for the development of adverse LV remodeling at 6 months following AMI. The main findings are as follows: (**1**) Adverse LV remodeling occurred in 11 of 64 (17%) AMI patients after a follow-up of 6 months. (**2**) At baseline, edema and infarct size were larger in AMI patients, who developed adverse LV remodeling, and ECV_infarct_ and native T2_infarct_ were markedly increased in this patient group. (**3**) AMI patients with adverse LV remodeling had a larger infarct size and reduced LVEF at 6-month follow-up compared patients without remodeling. (**4**) ECV_infarct_ and infarct size by LGE were the best predictors for adverse LV remodeling, and native T1_infarct_ was inferior to native T2_infarct_ and edema size by T2w to predict remodeling.

### Adverse LV remodeling

In our cohort, we adopted a definition of adverse LV remodeling as a progressive increase of indexed LVEDV of at least 20% at 6 months after AMI, and adverse LV remodeling occurred in 17% of patients. Wu et al defined adverse LV remodeling as an increase in indexed LVEDV of ≥ 20% at least 4 months from baseline, and a similar incidence of adverse LV remodeling (16%) was reported [24]. However, Carrick et al adopted the same definition for adverse LV remodeling as in the current study demonstrating that only 12% of AMI patients developed subsequent remodeling [25]. Adverse LV remodeling is multifactorial and involves multiple mechanisms. Some pathophysiologic conditions like hypertension [26], diabetes [27], valvular disease [28], and coronary microvascular dysfunction [29] also contribute to adverse LV remodeling. In our study, there was no difference between remodeling and non-remodeling patients in terms of hypertension and diabetes. In addition to infarct size, the excessive inflammatory response may be a major contributor to the development of adverse remodeling [30].

In the current study, AMI patients who developed adverse LV remodeling had larger edema size, infarct size, and higher ECV_infarct_ at baseline than subjects without LV remodeling, which was likely attributable to a more significant expansion of extracellular volume because of more severe edema and infiltration of inflammatory cells during the acute post-infarct period [8]. Namely, adverse LV remodeling may correspond to the excessive inflammatory response manifested by increased ECV. At follow-up, the cohort with adverse LV remodeling still had a larger infarct size and reduced LVEF, indicating a greater area of irreversibly injured myocardium. Further, in this study, peak CK exerted an acceptable predictive performance and patients, who developed LV remodeling, and had higher peak CK levels, which ordinarily reflect the spread and extent of damaged myocardium [31]. Hence, the severity of myocardial damage is likely associated with the development of an adverse LV dilatation.

### Prediction of adverse LV remodeling by contrast CMR

LGE imaging relies on the increased uptake of gadolinium-based contrast agents after AMI due to the increase in interstitial space caused by necrotic tissue and myocardial edema [32]. Infarct size measurement by LGE has excellent reproducibility both in acute and in chronic MI [7]. Through estimating infarct size by measuring LGE, the relationship between infarct size and adverse LV remodeling can be approached more directly [30]. We found that infarct size by LGE had a good predictive performance for adverse LV remodeling; further, a positive correlation was observed with the change in LVEDVi. This finding suggests that patients with larger infarct sizes had an obvious tendency to develop adverse LV remodeling. Previous studies were consistent with our finding that LV enlargement associated with adverse LV remodeling occurred in a strong linear relation with the initial infarct size [24, 33]. However, LGE at the early stage after infarction might overestimate infarct size due to severe edema [8].

ECV_infarct_ in this study showed the best capability of prospectively identifying patients at elevated risk of subsequent adverse LV remodeling. ECV_infarct_ potentially adds quantitative information about “infarct severity” for tissue disruption and loss of myocytes in the infarct area and complements LGE assessment as an additional predictor of LV functional recovery [34]. A prior study from Chen et al suggested that the severity of the myocardial injury can be mirrored by the increased extent of ECV, and more severe myocardial injury may promote adverse LV remodeling [14]. An experimental study based on a rat model supported the notion that the infarct area involves metabolic activities regulated by a complex array of cytokines, including the expression of multiple collagens and proteins in the extracellular matrix [35]. The CMR-derived ECV is a surrogate for all components of the myocardial interstitium, which includes not only collagen, but also non-collagenous extracellular matrix proteins, fibroblasts, vessels, and incompressible fluid [36]. Thus, one could speculate that patients with higher ECV_infarct_ might have particularly vulnerable components in the infarct tissue that predisposes to dilatation. However, further in vivo imaging combined with pathological validation is warranted to elucidate this hypothesis.

Previous studies demonstrated that ECV in the remote myocardium indicates the links between systemic inflammation and adverse LV remodeling after myocardial infarct [37, 38]. Our observation contradicts previous studies showing no significant differences regarding ECV_remote_ between patients with and without remodeling. The discrepancy among studies might relate to varying inflammation responses in the remote myocardium. Acutely interstitial alterations in the remote myocardium would be a dynamic reversible process [39]; CMR acquisition window may to some extent introduce heterogeneity.

### Prediction of adverse LV remodeling by non-contrast CMR

Myocardial T2 value remains one predominant index to assess myocardial edema, which may be elevated by alterations in myocardial water state (free water and bound water), even in the absence of an increase in net water content [40]. Some conditions are accompanied by elevated myocardial T2 even though the net water content is not altered. This might be the case when fluid shifts between cardiomyocytes and the interstitial compartment, when there is microstructural disruption, and in association with altered fluid composition during the different phases of edema evolution [41]. Importantly, quantitative T2 mapping overcomes several limitations of conventional T2w imaging and results in more accurate assessment of myocardial edema [41], thereof native T2 relaxation times may be more sensitive to reflect the changes in the molecular environment for the development of LV remodeling. As such, this study showed that native T2_infarct_ had a better predictive value than edema size by T2w imaging for the development of an adverse LV remodeling following AMI.

Carrick et al found that native T1 in the infarcted area was not associated with LV volumes at follow-up and there was no evidence of non-linearity between native T1 and LV outcomes [42]; our study confirmed an analogous finding that native T1_infarct_ had no significant correlation with the change in LVEDVi and had the lowest predictive value.

### Study limitations

The investigated cohort in this single-center study was relatively small, and may not represent the general AMI population. However, all patients were recruited consecutively according to the stringent selection criteria and were treated in accordance with the contemporary guidelines. Given the lack of CMR-derived data at different time points early after infarction, no further comparisons between previous work and the current data regarding the predictive performance for LV remodeling can be made. The CMR acquisition window might have had a potential influence on the results due to the dynamic changes. More future studies with a larger population and further subgroup analyses based on time window are needed to validate the association between ECV_remote_ and LV remodeling. In addition, the applications of artificial intelligence (AI) might result in faster image acquisition and improve the accuracy of myocardial quantification as already demonstrated by multiple studies [43]. The subsequent research in combination with an AI algorithm would potentially achieve higher accuracy rate in risk prediction for ventricular remodeling.

## Conclusions

This single-center study showed the development of adverse LV remodeling in 17% of AMI patients at 6-month follow-up. Patients with LV remodeling had larger edema and infarct sizes at baseline compared to patients without subsequent LV remodeling. Further, ECV and myocardial native T2 of infarcted myocardium were higher in the remodeling group at baseline. Also, AMI patients with adverse LV remodeling had a larger infarct size and reduced LVEF at 6-month follow-up. Importantly, it was shown that the development of adverse LV remodeling can be reliably predicted by ECV in the infarcted area at baseline and initial infarct size by LGE. Edema size by conventional T2w imaging and native T2 in the infarcted area also have a good predictive value, whereas native T1 constitutes a poor parameter to predict adverse LV remodeling.

### Supplementary Information

Below is the link to the electronic supplementary material.

Supplementary file1 (PDF 412 KB)

## Abbreviations

AHA: American Heart Association
AMI: Acute myocardial infarct
AUC: Area under the curve
BMI: Body mass index
BSA: Body surface area
CK-MB: Creatine kinase myocardial band
CMR: Cardiac magnetic resonance
ECV: Extracellular volume
EDVi: End-diastolic volume index
EF: Ejection fraction
ESVi: End-systolic volume index
LA: Left atrial
LGE: Late gadolinium enhancement
LV: Left ventricular
MOLLI: Modified look-locker inversion recovery
NSTEMI: Non-ST-segment elevation myocardial infarct
PPCI: Primary percutaneous coronary intervention
PSIR: Phase-sensitive inversion recovery
ROC: Receiver-operating characteristic
SSFP: Steady-state free-precession
STEMI: ST-segment elevation myocardial infarct
STIR: Suppressed triple inversion-recovery

## Declarations

### Guarantor

The scientific guarantor of this publication is Enver Tahir, MD.

### Statistics and Biometry

No complex statistical methods were necessary for this paper.

### Study subjects or cohorts overlap

The study population partly consisted of individuals, who had been evaluated in two previously published studies, one focused on the diagnostic accuracy of quantitative native T1 and T2 mapping compared to the assessment of edema on standard T2w imaging to differentiate acute from chronic MI [1], the other demonstrated the development of global and segmental myocardial strain in acute and chronic STEMI [2].

Tahir E, Sinn M, Bohnen S, Avanesov M, Säring D, Stehning C, Schnackenburg B, Eulenburg C, Wien J, Radunski UK, Blankenberg S, Adam G, Higgins CB, Saeed M, Muellerleile K, Lund GK. Acute versus Chronic Myocardial Infarction: Diagnostic Accuracy of Quantitative Native T1 and T2 Mapping versus Assessment of Edema on Standard T2-weighted Cardiovascular MR Images for Differentiation. Radiology. 2017 Oct;285(1):83–91. doi:10.1148/radiol.2017162338. Epub 2017 Jul 3. PMID: 28678672.

Erley J, Starekova J, Sinn M, Muellerleile K, Chen H, Harms P, Naimi L, Meyer M, Cavus E, Schneider J, Blankenberg S, Lund GK, Adam G, Tahir E. Cardiac magnetic resonance feature tracking global and segmental strain in acute and chronic ST-elevation myocardial infarction. Sci Rep. 2022 Dec 31;12(1):22644. doi:10.1038/s41598-022-26968-4. PMID: 36587037; PMCID: PMC9805431.

### Methodology

• prospective

• observational

• performed at one institution

## References

[1] Bolognese L, Neskovic AN, Parodi G (2002). Left ventricular remodeling after primary coronary angioplasty: patterns of left ventricular dilation and long-term prognostic implications. Circulation.

[2] Mitchell GF, Lamas GA, Vaughan DE, Pfeffer MA (1992). Left ventricular remodeling in the year after first anterior myocardial infarction: a quantitative analysis of contractile segment lengths and ventricular shape. J Am Coll Cardiol.

[3] Pfeffer MA (1995). Left ventricular remodeling after acute myocardial infarction. Annu Rev Med.

[4] Gaudron P, Eilles C, Kugler I, Ertl G (1993). Progressive left ventricular dysfunction and remodeling after myocardial infarction Potential mechanisms and early predictors. Circulation.

[5] Sinn MR, Lund GK, Muellerleile K (2021). Prognosis of early pre-discharge and late left ventricular dilatation by cardiac magnetic resonance imaging after acute myocardial infarction. Int J Cardiovasc Imaging.

[6] Hamirani YS, Wong A, Kramer CM, Salerno M (2014). Effect of microvascular obstruction and intramyocardial hemorrhage by CMR on LV remodeling and outcomes after myocardial infarction: a systematic review and meta-analysis. JACC Cardiovasc Imaging.

[7] Thiele H, Kappl MJ, Conradi S, Niebauer J, Hambrecht R, Schuler G (2006). Reproducibility of chronic and acute infarct size measurement by delayed enhancement-magnetic resonance imaging. J Am Coll Cardiol.

[8] Jablonowski R, Engblom H, Kanski M (2015). Contrast-enhanced CMR overestimates early myocardial infarct size: mechanistic insights using ECV measurements on day 1 and day 7. JACC Cardiovasc Imaging.

[9] Ugander M, Oki AJ, Hsu LY (2012). Extracellular volume imaging by magnetic resonance imaging provides insights into overt and sub-clinical myocardial pathology. Eur Heart J.

[10] Reindl M, Eitel I, Reinstadler SJ (2020) Role of cardiac magnetic resonance to improve risk prediction following acute ST-elevation myocardial infarction. J Clin Med 9:1041 10.3390/jcm9041041 PMC7231095 32272692

[11] Verhaert D, Thavendiranathan P, Giri S (2011). Direct T2 quantification of myocardial edema in acute ischemic injury. JACC Cardiovasc Imaging.

[12] Layland J, Rauhalammi S, Lee MM et al (2017) Diagnostic accuracy of 3.0-T magnetic resonance T1 and T2 mapping and T2-weighted dark-blood imaging for the infarct-related coronary artery in non-ST-segment elevation myocardial infarction. J Am Heart Assoc 6:e004759 10.1161/JAHA.116.004759 PMC5532996 28364045

[13] Tahir E, Sinn M, Bohnen S et al (2017) Acute versus chronic myocardial infarction: diagnostic accuracy of quantitative native T1 and T2 mapping versus assessment of edema on standard T2-weighted cardiovascular MR images for differentiation. Radiology 285(1):83–91. 10.1148/radiol.2017162338 10.1148/radiol.2017162338 28678672

[14] Chen BH, An DA, He J, Xu JR, Wu LM, Pu J (2020). Myocardial extracellular volume fraction allows differentiation of reversible versus irreversible myocardial damage and prediction of adverse left ventricular remodeling of ST-elevation myocardial infarction. J Magn Reson Imaging.

[15] Thygesen K, Alpert JS, Jaffe AS (2012). Third universal definition of myocardial infarction. Circulation.

[16] Erley J, Starekova J, Sinn M et al (2022) Cardiac magnetic resonance feature tracking global and segmental strain in acute and chronic ST-elevation myocardial infarction. Sci Rep 12(1):22644. 10.1038/s41598-022-26968-4 10.1038/s41598-022-26968-4 PMC9805431 36587037

[17] Messroghli DR, Greiser A, Frohlich M, Dietz R, Schulz-Menger J (2007). Optimization and validation of a fully-integrated pulse sequence for modified look-locker inversion-recovery (MOLLI) T1 mapping of the heart. J Magn Reson Imaging.

[18] Baessler B, Schaarschmidt F, Stehning C, Schnackenburg B, Maintz D, Bunck AC (2015). A systematic evaluation of three different cardiac T2-mapping sequences at 1.5 and 3T in healthy volunteers. Eur J Radiol.

[19] Säring D, Ehrhardt J, Stork A, Bansmann MP, Lund GK, Handels H (2006). Computer-assisted analysis of 4D cardiac MR image sequences after myocardial infarction. Methods Inf Med.

[20] Schulz-Menger J, Bluemke DA, Bremerich J (2020). Standardized image interpretation and post-processing in cardiovascular magnetic resonance - 2020 update : Society for Cardiovascular Magnetic Resonance (SCMR): Board of Trustees Task Force on Standardized Post-Processing. J Cardiovasc Magn Reson.

[21] White SK, Sado DM, Fontana M (2013). T1 mapping for myocardial extracellular volume measurement by CMR: bolus only versus primed infusion technique. JACC Cardiovasc Imaging.

[22] Dall'Armellina E, Karia N, Lindsay AC (2011). Dynamic changes of edema and late gadolinium enhancement after acute myocardial infarction and their relationship to functional recovery and salvage index. Circ Cardiovasc Imaging.

[23] Hamshere S, Jones DA, Pellaton C (2016). Cardiovascular magnetic resonance imaging of myocardial oedema following acute myocardial infarction: is whole heart coverage necessary?. J Cardiovasc Magn Reson.

[24] Wu E, Ortiz JT, Tejedor P (2008). Infarct size by contrast enhanced cardiac magnetic resonance is a stronger predictor of outcomes than left ventricular ejection fraction or end-systolic volume index: prospective cohort study. Heart.

[25] Carrick D, Haig C, Rauhalammi S (2015). Pathophysiology of LV remodeling in survivors of STEMI: inflammation, remote myocardium, and prognosis. JACC Cardiovasc Imaging.

[26] Yildiz M, Oktay AA, Stewart MH, Milani RV, Ventura HO, Lavie CJ (2020). Left ventricular hypertrophy and hypertension. Prog Cardiovasc Dis.

[27] Levelt E, Mahmod M, Piechnik SK (2016). Relationship between left ventricular structural and metabolic remodeling in type 2 diabetes. Diabetes.

[28] Yarbrough WM, Mukherjee R, Ikonomidis JS, Zile MR, Spinale FG (2012). Myocardial remodeling with aortic stenosis and after aortic valve replacement: mechanisms and future prognostic implications. J Thorac Cardiovasc Surg.

[29] Park SM, Wei J, Cook-Wiens G (2019). Left ventricular concentric remodelling and functional impairment in women with ischaemia with no obstructive coronary artery disease and intermediate coronary flow reserve: a report from the WISE-CVD study. Eur Heart J Cardiovasc Imaging.

[30] Westman PC, Lipinski MJ, Luger D (2016). Inflammation as a driver of adverse left ventricular remodeling after acute myocardial infarction. J Am Coll Cardiol.

[31] Hashimoto Y, Soeda T, Seno A (2022). Reverse remodeling and non-contrast T1 hypointense infarct core in patients with reperfused acute myocardial infarction. Circ J.

[32] Mather AN, Fairbairn TA, Artis NJ, Greenwood JP, Plein S (2011). Timing of cardiovascular MR imaging after acute myocardial infarction: effect on estimates of infarct characteristics and prediction of late ventricular remodeling. Radiology.

[33] Lund GK, Stork A, Muellerleile K (2007). Prediction of left ventricular remodeling and analysis of infarct resorption in patients with reperfused myocardial infarcts by using contrast-enhanced MR imaging. Radiology.

[34] Kidambi A, Motwani M, Uddin A (2017). Myocardial extracellular volume estimation by CMR predicts functional recovery following acute MI. JACC Cardiovasc Imaging.

[35] Deten A, Holzl A, Leicht M, Barth W, Zimmer HG (2001). Changes in extracellular matrix and in transforming growth factor beta isoforms after coronary artery ligation in rats. J Mol Cell Cardiol.

[36] Nakamori S, Dohi K, Ishida M (2018). Native T1 mapping and extracellular volume mapping for the assessment of diffuse myocardial fibrosis in dilated cardiomyopathy. JACC Cardiovasc Imaging.

[37] Bulluck H, Rosmini S, Abdel-Gadir A et al (2016) Automated extracellular volume fraction mapping provides insights into the pathophysiology of left ventricular remodeling post-reperfused ST-elevation myocardial infarction. J Am Heart Assoc 5:e003555 10.1161/JAHA.116.003555 PMC5015393 27402229

[38] Ishiyama M, Kurita T, Nakamura S (2021). Prognostic importance of acute phase extracellular volume evaluated by cardiac magnetic resonance imaging for patients with acute myocardial infarction. Int J Cardiovasc Imaging.

[39] Chan W, Duffy SJ, White DA (2012). Acute left ventricular remodeling following myocardial infarction: coupling of regional healing with remote extracellular matrix expansion. JACC Cardiovasc Imaging.

[40] Friedrich MG (2010). Myocardial edema–a new clinical entity?. Nat Rev Cardiol.

[41] O'Brien AT, Gil KE, Varghese J, Simonetti OP, Zareba KM (2022). T2 mapping in myocardial disease: a comprehensive review. J Cardiovasc Magn Reson.

[42] Carrick D, Haig C, Rauhalammi S (2016). Prognostic significance of infarct core pathology revealed by quantitative non-contrast in comparison with contrast cardiac magnetic resonance imaging in reperfused ST-elevation myocardial infarction survivors. Eur Heart J.

[43] Lanzafame LRM, Bucolo GM, Muscogiuri G et al (2023) Artificial intelligence in cardiovascular CT and MR imaging. Life (Basel) 13:507 10.3390/life13020507 PMC9968221 36836864

