# Supplementary material for: Contrast-enhanced cardiac MRI is superior to non-contrast mapping to predict left ventricular remodeling at 6 months after acute myocardial infarction
Source: Eur Radiol. 2023 Sep 4;34(3):1863–74. doi: 10.1007/s00330-023-10100-9 (PMC10873445; doi:10.1007/s00330-023-10100-9)

Table S1: Receiver-operating characteristic curve analysis of CMR parameters of the involved segments at baseline to predict adverse LV remodeling at 6-month follow-up.

Parameters	AUC (95% CI)	*P*-value*	Cut-off	TP	FN	FP	TN	Total	Sensitivity (%)	Specificity (%)	Accuracy (%)	PPV (%)	NPV (%)
ECV	0.653 (0.601-0.603)	**<0.01**	>34 %	36	4	175	136	351	90 (76-97)	44 (38-49)	49 (44-54)	17 (13-23)	97 (93-99)
Infarct size	0.566 (0.510-0.622)	0.105	>25 %	43	26	119	129	317	62 (51-73)	52 (46-58)	54 (49-60)	27 (20-34)	83 (77-88)
Native T2	0.686 (0.635-0.744)	**<0.01**	>66 ms	36	13	117	189	355	73 (60-84)	62 (56-67)	63 (58-68)	24 (17-31)	94 (89-96)
Edema size	0.545 (0.497-0.593)	0.204	>31 %	58	24	210	132	424	71 (60-80)	39 (34-44)	45 (40-50)	22 (17-27)	85 (78-90)
Native T1	0.561 (0.508-0.614)	0.191	>1154 ms	28	13	160	155	356	68 (53-81)	49 (44-55)	51 (46-57)	15 (10-21)	92 (87-96)

***** For statistical significance of AUCs.
Abbreviations: AUC, area under the curve; CI, confidence interval; ECV, extracellular volume; FN, false negative; FP, false positive; NPV, negative predictive value; PPV, positive predictive value; TN, true negative; TP, true positive.

Figure S1: Schematic representation of the involved segments with the respective infarcted area (%LV) in patients with (top) and without (bottom) adverse LV remodeling according to the AHA 16-segment model. AHA, American Heart Association; LV, left ventricular.

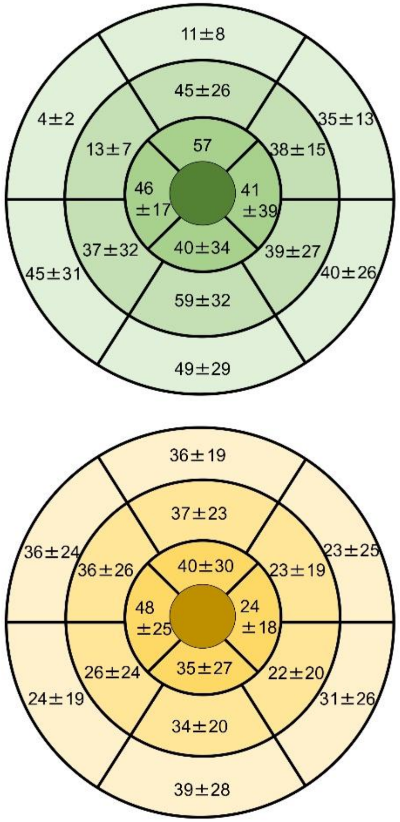

Figure S2: Schematic representation of the involved segments with the respective edematous area (%LV) in patients with (top) and without (bottom) adverse LV remodeling according to the AHA 16-segment model. AHA, American Heart Association; LV, left ventricular.

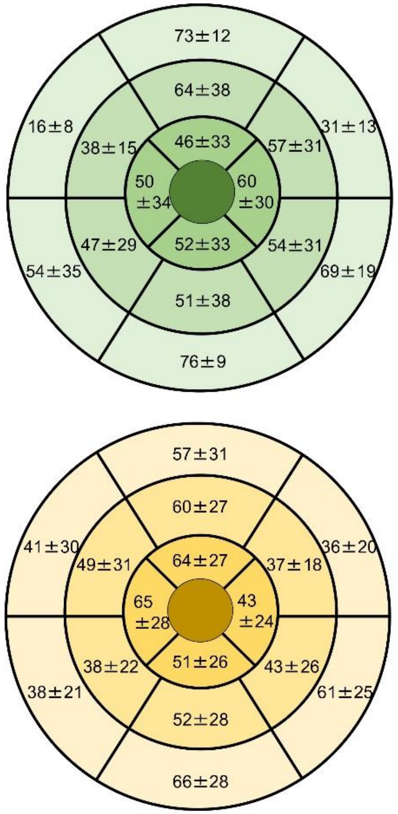

Figure S3: Schematic representation of the involved segments with the respective average native T2 relaxation times (ms) in patients with (top) and without (bottom) adverse LV remodeling according to the AHA 16-segment model. AHA, American Heart Association; LV, left ventricular.

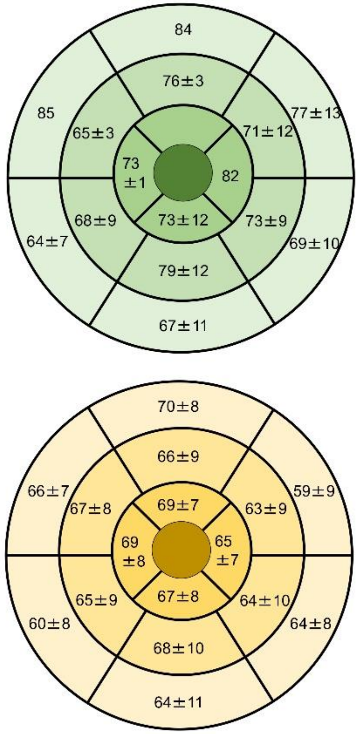

Figure S4: Schematic representation of the involved segments with the respective average native T1 relaxation times (ms) in patients with (top) and without (bottom) adverse LV remodeling according to the AHA 16-segment model. AHA, American Heart Association; LV, left ventricular.

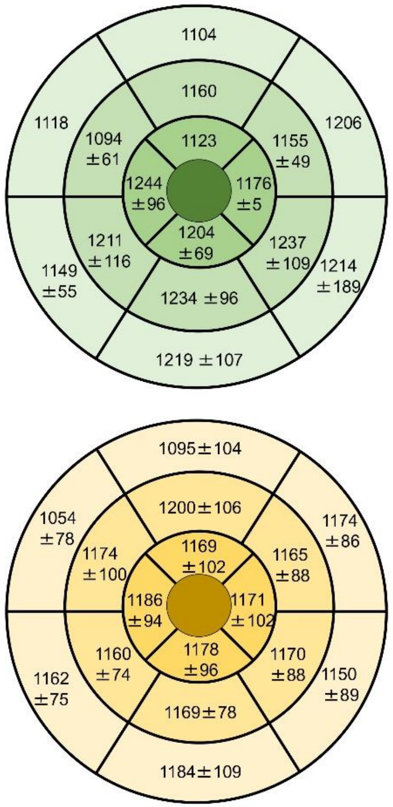

Figure S5: Schematic representation of the involved segments with the respective average ECV (%) in patients with (top) and without (bottom) adverse LV remodeling according to the AHA 16-segment model. AHA, American Heart Association; ECV, extracellular volume; LV, left ventricular.

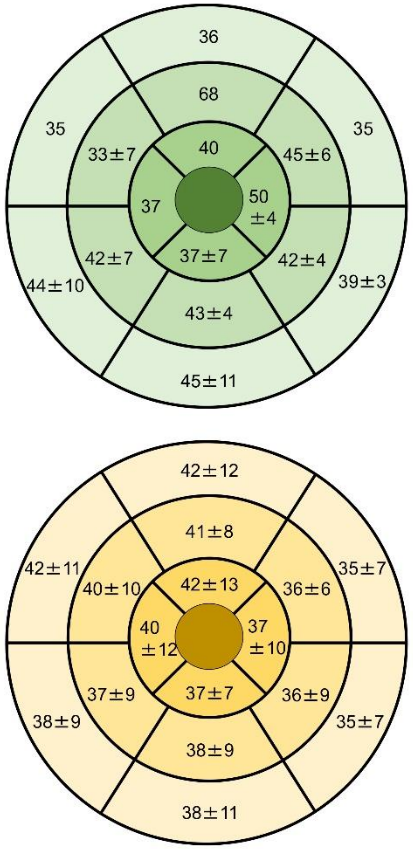

Supplement: Supplementary file 1 — Supplementary file1 (PDF 412 KB) [file 330_2023_10100_MOESM1_ESM.pdf]
